# Phenylalanine Metabolism Regulates Reproduction and Parasite Melanization in the Malaria Mosquito

**DOI:** 10.1371/journal.pone.0084865

**Published:** 2014-01-07

**Authors:** Silke Fuchs, Volker Behrends, Jacob G. Bundy, Andrea Crisanti, Tony Nolan

**Affiliations:** 1 Department of Life Sciences, Imperial College London, London, United Kingdom; 2 Department of Surgery and Cancer, Imperial College London, London, United Kingdom; Institut national de la santé et de la recherche médicale - Institut Cochin, France

## Abstract

The blood meal of the female malaria mosquito is a pre-requisite to egg production and also represents the transmission route for the malaria parasite. The proper and rapid assimilation of proteins and nutrients in the blood meal creates a significant metabolic challenge for the mosquito. To better understand this process we generated a global profile of metabolite changes in response to blood meal of *Anopheles gambiae*, using Gas Chromatography-Mass Spectrometry (GC-MS). To disrupt a key pathway of amino acid metabolism we silenced the gene phenylalanine hydroxylase (*PAH*) involved in the conversion of the amino acid phenylalanine into tyrosine. We observed increased levels of phenylalanine and the potentially toxic metabolites phenylpyruvate and phenyllactate as well as a reduction in the amount of tyrosine available for melanin synthesis. This in turn resulted in a significant impairment of the melanotic encapsulation response against the rodent malaria parasite *Plasmodium berghei*. Furthermore silencing of *PAH* resulted in a significant impairment of mosquito fertility associated with reduction of laid eggs, retarded vitellogenesis and impaired melanisation of the chorion. Carbidopa, an inhibitor of the downstream enzyme DOPA decarboxylase that coverts DOPA into dopamine, produced similar effects on egg melanization and hatching rate suggesting that egg chorion maturation is mainly regulated via dopamine. This study sheds new light on the role of amino acid metabolism in regulating reproduction and immunity.

## Introduction

Female *Anopheles* mosquitoes require a blood meal of their human or animal hosts in order to initiate egg development. Repeated blood meals increase not only the reproductive capacity but also make those females efficient disease vectors of malaria by increasing the potential to spread *Plasmodium* parasites from host to host. Because of this tight link between reproduction and disease transmission an understanding of the molecular mechanisms that control the reproduction biology and immunity response of these vectors could elucidate new ways to block parasite transmission.

Directly after taking a blood meal a tightly regulated amino acid metabolism is essential on three fronts: oogenesis [1]; innate immune response [2]; preventing accumulation of toxic levels of amino acid metabolites [3]. In oogenesis, ingested proteins are broken down into amino acids that signal through the target of rapamycin (TOR) pathway the synthesis of yolk protein precursors in the fat body that are deposited into developing oocytes during vitellogenesis [4], [5]. Infusion of a balanced cocktail of amino acids is sufficient to induce vitellogenesis in *Aedes aegypti* mosquitoes [1], [6] and more recent work has shown that the presence of up to 17 amino acids is sufficient in triggering this process [7], [8], [9]. Metabolites of specific amino acids are also critical for the formation and maturation of the egg chorion. Tyrosine, either ingested directly or formed through hydroxylation of ingested phenylalanine by phenylalanine hydroxylase (PAH), is considered a rate-determining factor in the melanization reaction that is responsible for chorion hardening [10], [11]. Tyrosine is hydroxylated to form 3,4 dihydroxyphenylalanine (DOPA) which is in turn converted into dopamine by DOPA decarboxylase (DDC). Both DOPA and dopamine can be converted to DOPA-melanin or dopamine-melanin, respectively, by a range of enzymes termed prophenoloxidases (PPO) [11], [12]. The same PPO enzymes involved in egg hardening have also been shown as essential in the innate immune response against a wide range of mosquito pathogens [2], [13], [14], [15], [16], [17].

In addition to being required for protein synthesis several amino acids and their direct metabolites also function as neurotransmitters [18], [19], [20]. Dopamine is not only the precursor to melanin; it is also a potent neurotransmitter active in dopaminergic neurons across a wide range of animals and must be tightly regulated. In vertebrates, in addition to disturbing the neurotransmitter equilibrium, mis-regulation of the conversion of amino acid precursors such as phenylalanine and tyrosine, through mutations in the enzymes PAH or DDC, into dopamine can lead to accumulation of toxic levels of these amino acids or their metabolites, often resulting in behavioural defects and reduced lifespan [3], [21].

Given a potential role for phenylalanine metabolism in life history traits of *Anopheles gambiae* such as egg production, immunity, behaviour and lifespan that are relevant to its capacity to transmit disease, we focused on perturbing phenylalanine metabolism. Here, using RNAi knockdown to target the first enzyme of this pathway, PAH, we used a Gas Chromatography- Mass spectrometry (GC-MS)-based metabolic profiling approach to quantify changes in amino acids and other metabolites post blood meal and to shed light on the pathways employed by the mosquito in assimilation of the blood meal.

## Results

### Metabolic Profiling of the Phenylalanine Pathway in Response to Blood Meal and PAH Knockdown

**Figure 1:**
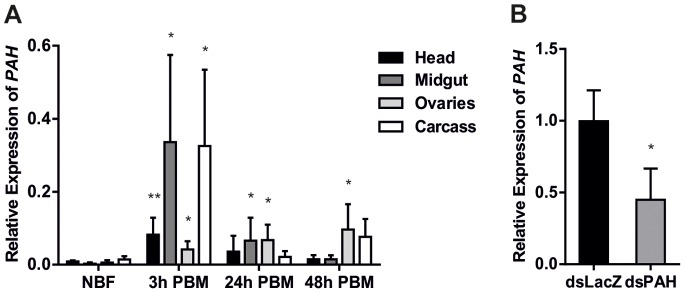
Transcriptional analysis of *PAH* in various tissues and following dsRNA injection. **A)** Q-PCR determination of *PAH* mRNA levels in the head, midgut, ovaries and remaining carcass of *A. gambiae* females in response to blood meal. Pools of 3 females were dissected and their total RNA extracted at a non-blood-fed (NBF) stage as well as 3 h, 24 h and 48 h post-blood meal (PBM). *PAH* transcript abundance is represented as mean proportion ± SD of the expression of the ribosomal protein gene *RPL19* of 3 independent biological repeats (t-test, *p<0.05, **p<0.01). **B)** 24 h PBM *PAH* expression was down-regulated in ds*PAH* injected females compared to ds*LacZ* injected controls. Transcript abundance was standardized to *RPL19* and represented as the mean proportion ± SD of the expression recorded in the *LacZ* control of 3 independent biological repeats (t-test,*p<0.05).

**Figure 2:**
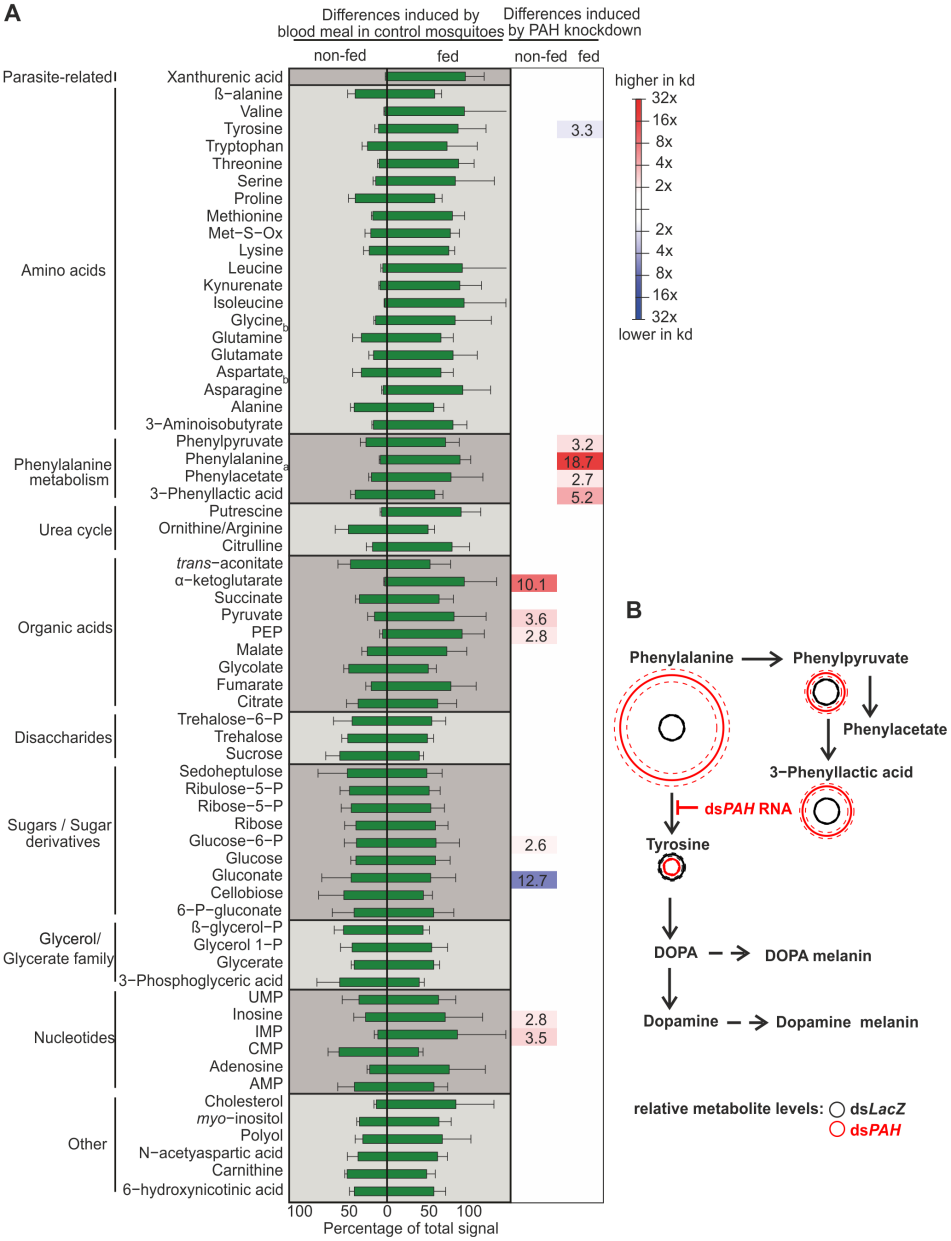
GC-MS mosquito metabolome in response to PAH knockdown. **A)** Metabolites were extracted from 2 females and pooled for GC-MS analysis. The green bars represent the mean percentage of total metabolite signal ± SD in fed and non-fed *dsLacZ* injected controls from 4 independent biological repeats. The heatmap represents the mean fold change in metabolite signal in non-fed and fed dsPAH injected mosquitoes compared to the respective non-fed and fed *dsLacZ* injected controls. ^a^putative metabolite identity (poor signal to noise), ^b^labile metabolite, therefore only approximate quantification. **B)** Targeted analysis of GC-MS detectable metabolites affected by PAH knockdown within the phenylalanine metabolism pathway. 24 h post-blood meal aqueous metabolites were extracted from 2 blood-fed females injected with ds*PAH* or ds*LacZ* and pooled for analysis. The circles represent the mean metabolite levels (thick line) ± SD (dashed line) of ds*PAH* females relative to the metabolite levels of the dsLacZ controls.

We investigated the transcription profile of the gene *PAH* in response to blood feeding in different tissues and organs. The relative mRNA levels of the putative *A. gambiae PAH* gene (AGAP005712) were measured using qPCR in head, midgut, ovaries and remaining carcass at different time points from 3 to 48 hours after blood feeding. This analysis revealed that blood feeding induced important transcriptional changes of *PAH* in all tissues examined (p<0.05) **(** **Figure 1A** **)**. At 3 h post-blood meal (PBM) the mRNA was mainly transcribed in the head, carcass and midgut while the highest level of expression was observed in the ovaries at 24 h PBM. The spatial-temporal expression pattern of *PAH* mirrored transcriptional changes associated with blood meal induced metabolic and physiological changes ranging from immunity-related responses involving the fatbody, midgut and hemocytes [22], [23], protein digestion in the midgut [24], the synthesis of neuropeptides and hormones in the head [25] and egg development in the ovaries [26]. To silence the *PAH* gene we designed dsRNA (ds*PAH*) targeting a region common to all three PAH protein isoforms **(Figure S1)** in blood-fed females. Quantification of gene transcript by qPCR revealed that *PAH* mRNA was significantly reduced (60%) in ds*PAH*-injected mosquitoes compared to a control group injected with dsRNA against a non-related bacterial gene *LacZ* (ds*LacZ*) (p<0.05) **(** **Figure 1B** **)**. To assess the effect of *PAH*-silencing we analyzed the metabolome of the mosquito using GC-MS. This analysis showed that in response to blood feeding the levels of amino acids, organic acids, nucleotides and other compounds such as cholesterol increased while disaccharides, sugars and glycerol levels remained constant **(** **Figure 2A** **)**. In *PAH*-silenced non-blood-fed insects we observed a marked increase in phosphoenolpyruvate (PEP), pyruvate and α-ketoglutarate, indicating a change in lower glycolysis and potentially assimilatory nitrogen metabolism. In response to blood-feeding, the most significant effect caused by PAH knockdown was the accumulation of phenylalanine (19 fold), phenyllactate (5 fold), phenylacetate (3 fold) and phenylpyruvate (3 fold) associated with a concomitant reduction of the tyrosine level (3 fold). We concluded that the observed reduction of *PAH* mRNA was sufficient to impair the conversion from phenylalanine to tyrosine. A more targeted GC-MS analysis specifically for known phenylalanine metabolites (allowing longer dwell time on the quantification ions) confirmed our initial findings in revealing increased levels of phenylpyruvate, phenylacetate and phenyllactate indicating that excess of phenylalanine is converted into these potentially toxic secondary metabolites **(** **Figure 2B** **)**. We were unable to determine mosquito neurotransmitter levels due to these being below the limit of quantification for the sampling/analysis approach used.

### Life History Phenotype of *PAH*-silenced Mosquitoes

**Figure 3:**
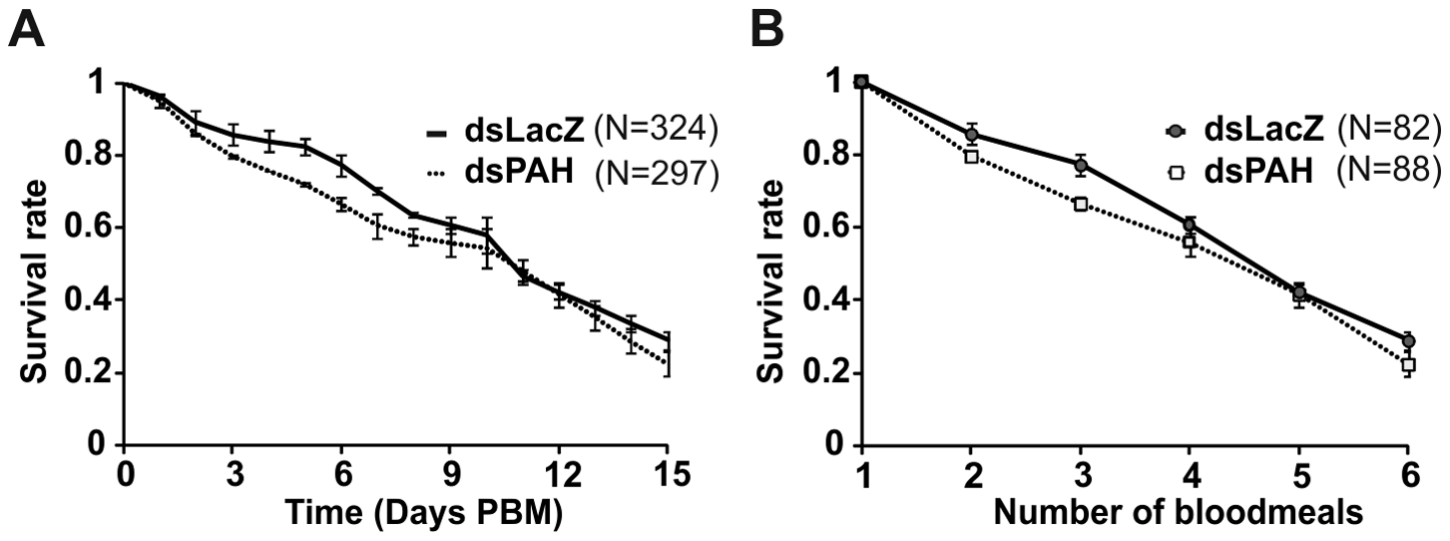
Reduced PAH activity does not decrease the survival of adult blood-fed mosquitoes. Mean survival ± SEM of ds*PAH* females and ds*LacZ* injected controls **A)** after a single blood meal of 6 independent experiments (PBM- time post-blood meal) (log-rank test, p>0.05) **B)** in response to multiple blood meals (time of blood meal is indicated by circles) of 2 independent experiments (log-rank test, p>0.05).

**Figure 4:**
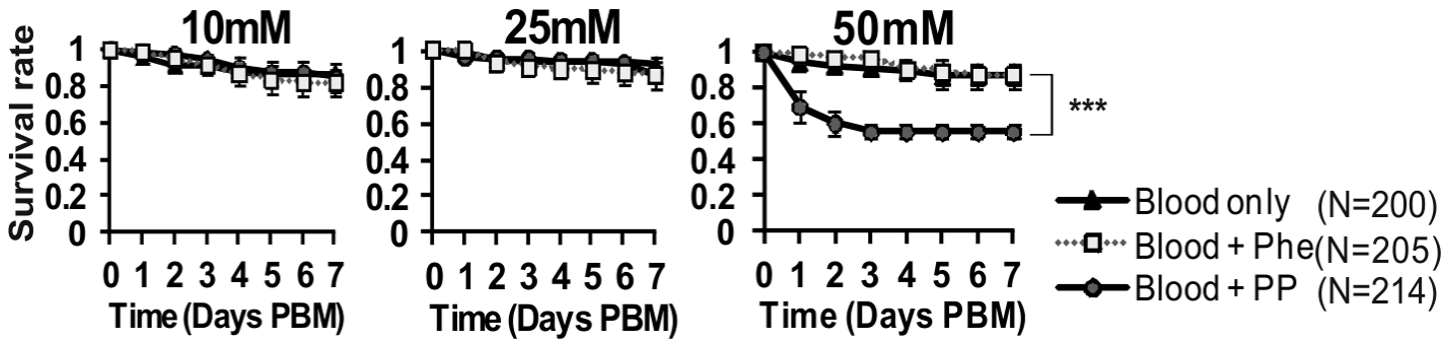
Large amounts of ingested phenylpyruvate are required to decrease the survival of adult *A. gambiae* mosquitoes. Females were fed on naive blood or blood supplemented with phenylpyruvate (PP) or phenylalanine (Phe) at a concentration of 10–50 mM and their survival was recorded daily until 7 days post blood meal (PBM). Combined survival data from three independent experiments are displayed as mean ± SEM (log-rank test, ***p<0.001).

In humans, mutations that inactivate the *PAH* gene are associated with a disease known as phenylketonuria (PKU), characterized by the accumulation of the toxic metabolites of phenylalanine, phenylpyruvate and phenyllactate, that are responsible for severe neurologic anomalies and premature death [3]. In female mosquitoes *PAH*-silencing caused a marked increase in the levels of phenylpyruvate and phenyllactate but we could detect neither obvious behavioural anomalies nor a significant reduction in survival **(** **Figure 3A** **)** even after multiple blood meals **(** **Figure 3B** **).** These findings would suggest that the metabolites are either not toxic or are not accumulated in sufficient amount to exert a biological effect. We also fed increasing amount of phenylpyruvate to female mosquitoes in an attempt to identify the conditions that could mimic the human disease in the mosquito. This analysis showed that when fed at a dose able to induce PKU in a rodent model (50 mM) [27], phenylpyruvate significantly reduced the life span of mosquitoes (p<0.001), indicating that the level at which this compound exert a toxic effect on female mosquito was not reached by *PAH*-silencing **(** **Figure 4** **)**.

### PAH Activity is Required for the Melanization Response against *P. berghei*

**Figure 5:**
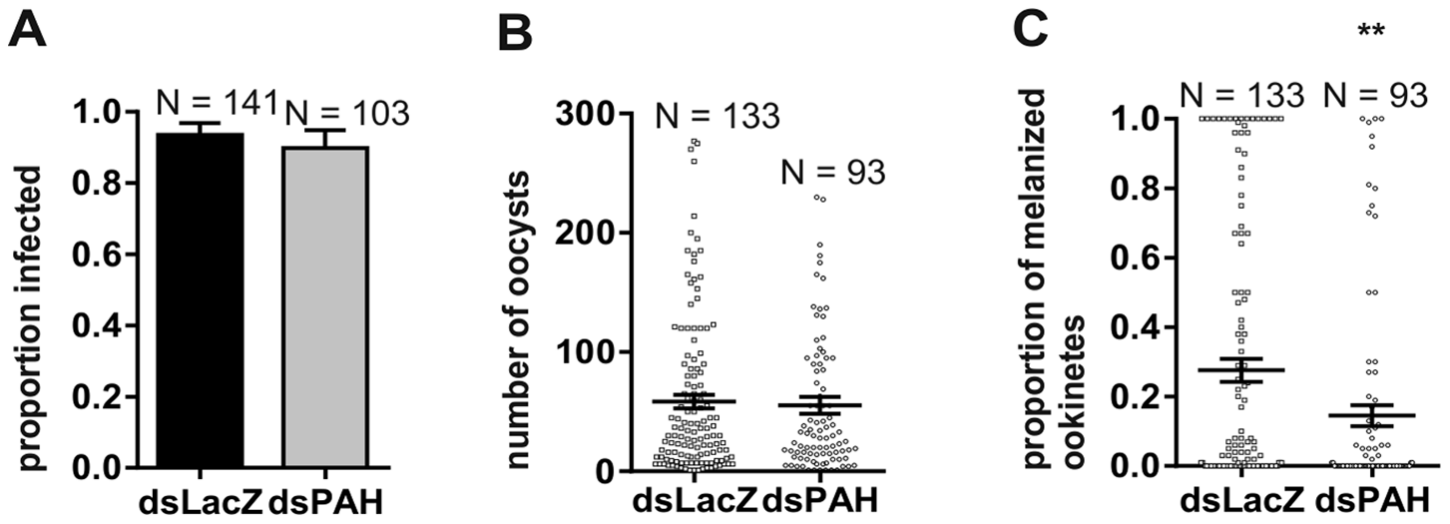
Knockdown of PAH causes a reduced melanization of *P. berghei* ookinetes. In 3 independent biological repeats ds*PAH-* and ds*LacZ*-injected (control) females were fed on a mouse infected with *P. berghei* parasites. Mosquito midguts were dissected and examined for oocysts 8 days after infection. **A)** Displayed is the proportion of females which harboured at least 1 oocyst (Likelihood of Infection: Fisher’s exact test, p>0.05) **B)** Oocyst load of ds*PAH* and ds*LacZ* injected females. The bars represent the mean ± SEM (Mann-Whitney U test, p>0.05). Only females with at least 1 oocyst and/or melanized ookinete were included in the analysis. **C)** Represented is the proportion of melanized ookinetes to the total number of oocysts per ds*PAH* or ds*LacZ* injected female. The bars indicate the mean ± SEM (t-test of arc-sine square root transformed proportion, **p<0.01).

Tyrosine is the rate-limiting substrate for the formation of melanin in response to immunological stimuli elicited in the mosquito by bacteria and microfilariae [2], [28], [29], [30]. The phenylalanine hydroxylase enzyme PAH, which produces the only endogenous source of tyrosine is up-regulated in *A. gambiae* hemocytes in response to malaria parasite infection [31] yet its involvement in melanotic encapsulation response has not been established in this species [32]. We investigated whether the limited availability of tyrosine caused by *PAH*-silencing had an impact on the ability of *A. gambiae* mosquitoes to encapsulate and melanize *P. berghei* ookinetes. We observed in both *PAH*-silenced and control injected mosquitoes a high prevalence of infected females (90–94%, p>0.05) carrying at least one *P. berghei* oocyst **(** **Figure 5A** **)**. We detected a high variability in the intensity of infection in the two groups and no significant difference was found (p>0.05) **(** **Figure 5B** **)**. However, when we investigated the melanization response we observed a significant reduction in the proportion of melanized ookinetes in ds*PAH* compared to ds*LacZ* injected mosquitoes (15% vs. 28% melanized, p<0.01) **(** **Figure 5C** **)**.

### PAH Activity Regulates Female Fertility

**Figure 6:**
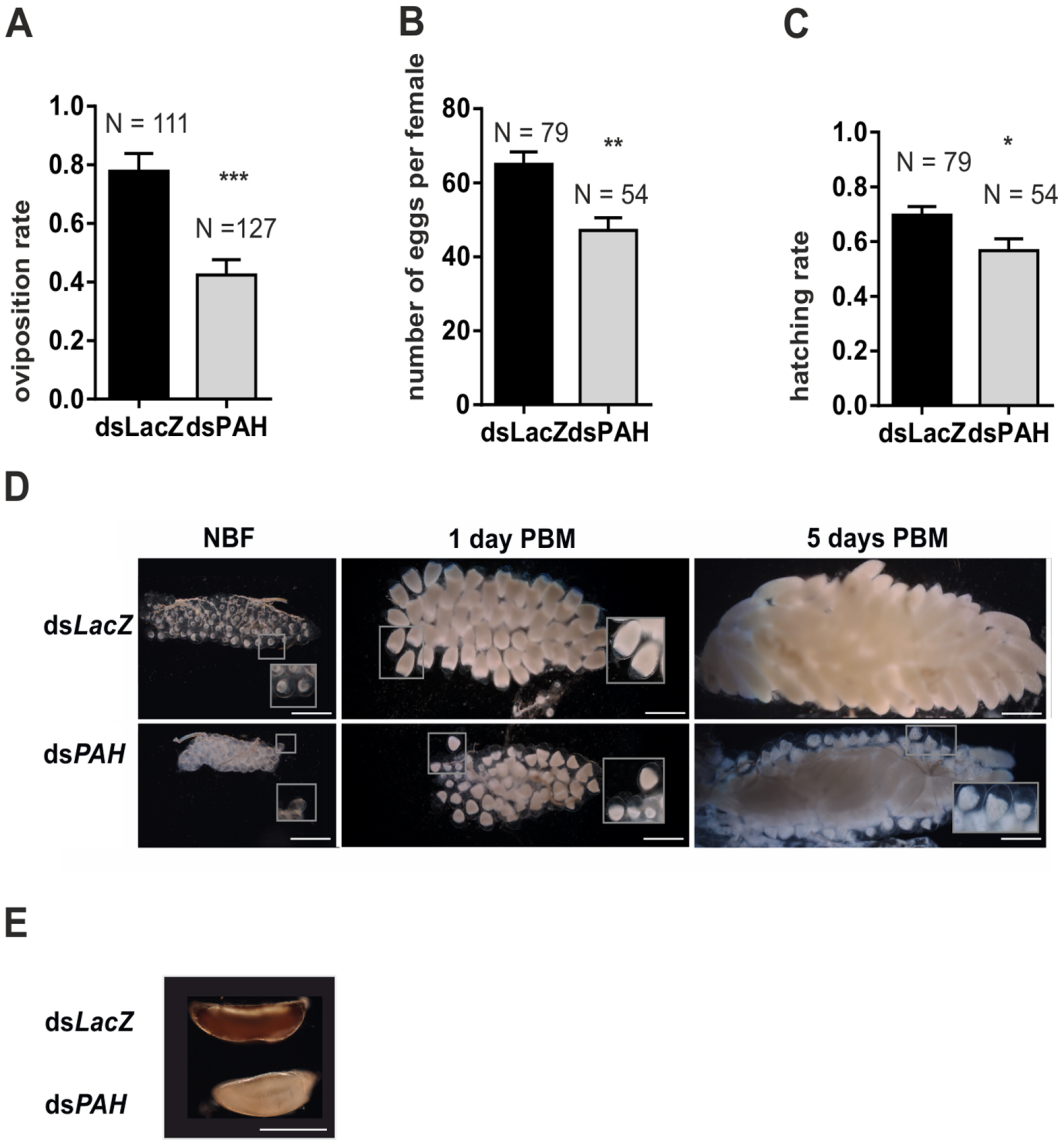
PAH knockdown leads to reduced fertility of *A. gambiae* mosquitoes. **A)** The mean proportion ±SEM of ds*PAH* and ds*LacZ* (control) injected females that oviposited (Likelihood of oviposition: Fisher’s exact test, ***p<0.001). **B)** Mean number ± SEM of eggs per ovipositing female injected with either ds*LacZ* or ds*PAH*. Only females that oviposited 1 egg or more were included in the analysis (t-test, **p<0.01). **C)** Mean ± SEM hatching rate of ds*PAH* and ds*LacZ* control injected females (t-test of arc-sine square root transformed proportion, *p<0.05). **D)** Upon dissection (N = 6 per time point) we observed that ovaries of ds*PAH* injected females were smaller. Females which did not oviposit contained a large fraction of undeveloped eggs in their ovaries 5 days post blood meal. Scale bar: 400 µm. **E)** When females were placed into water-filled oviposition cups to lay eggs, we observed unmelanized eggs 24 h post-oviposition laid by ds*PAH* injected females, indicating a malfunctioning melanin biosynthesis. Scale bar: 400 µm.

*A. gambiae* eggs are laid on the water surface and are sensitive to desiccation [33], [34]. Mosquito egg survival in the environment is dependent on the rapid maturation of the chorion that undergoes a tanning process associated with deposition of melanin and protein cross-linking shortly after oviposition [10]. The up-regulated expression of PAH in the ovaries at the time of egg formation and oviposition suggests a potential role of this gene in yolk protein synthesis and/or chorion maturation. We measured fertility and fecundity of individual *PAH*-silenced females compared to the respective ds*LacZ*- injected controls to investigate the role of this gene in egg maturation. Our results showed that only 42% of *PAH*-silenced females laid eggs compared to 78% of the control (p<0.001) **(** **Figure 6A** **)**. Those females that laid eggs showed a marked reduction in the number of viable progeny due to a combined effect on the reduced number of eggs laid per mosquito, (by 30%, p<0.01) **(** **Figure 6B** **)** and lower hatching rates (by 17%, p<0.05) **(** **Figure 6C** **)**. To assess whether failure to lay eggs was due to an arrest in the ovary maturation process we dissected *PAH*-silenced and control females five days after blood- feeding. In ds*PAH*-injected females we observed eggs that had not completed the maturation process with significantly reduced yolk, resembling those seen in control mosquitoes at day 1 post-feeding **(** **Figure 6D** **),** thus demonstrating the importance of PAH for mosquito egg development. Moreover, even in the non-blood-fed condition *PAH*-silenced mosquitoes contained significantly smaller ovarioles, suggesting a role for PAH also in the pre-vitellogenic maintenance of ovariole growth.

**Figure 7:**
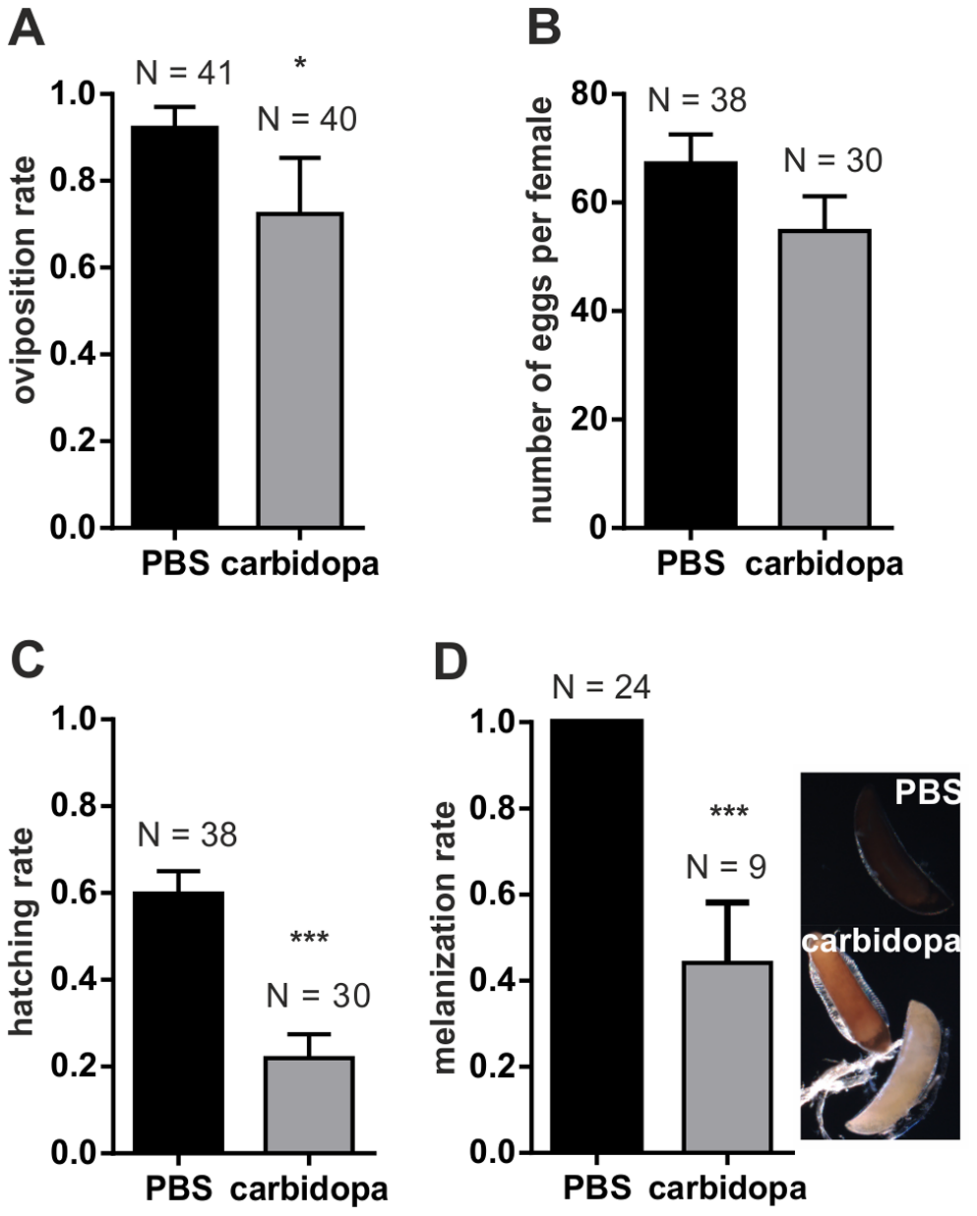
Injection of carbidopa caused reduced egg viability and melanization in *A. gambiae*. **A)** The mean proportion ±SEM of PBS (control) and carbidopa injected females that oviposited (Likelihood of oviposition: Fisher’s exact test, *p<0.05). **B)** Mean number ± SEM of eggs per ovipositing female injected with either PBS or carbidopa. (t-test, p>0.05). **C)** Mean ± SEM hatching rate of PBS and carbidopa injected females (t-test of arc-sine transformed proportion, ***p<0.001). Data were combined from 4 independent experiments. **D)** In response to carbidopa injection we observed a large proportion of light and unmelanized eggs. Displayed are the mean ± SEM melanization rate of eggs laid by PBS or carbidopa injected females from 2 independent experiments (t-test of arc-sine square root transformed proportion, ***p<0.001).

We also observed one day after oviposition that a substantial fraction of eggs laid by ds*PAH*-injected females aborted prior to full melanization of the chorion and appeared fragile and fragmented, a phenotype potentially caused by a lack of tyrosine oxidation products DOPA and dopamine that are downstream of PAH and that are required for melanin synthesis **(** **Figure 6E** **)**. To investigate this latter hypothesis we injected carbidopa [35], a synthetic inhibitor of the enzyme DOPA decarboxylase (DDC), an enzyme that would normally decarboxylate DOPA into dopamine. Carbidopa-injected mosquitoes showed a significant drop in oviposition rate (p<0.05) **(** **Figure 7A** **)**, embryo hatching rate (p<0.001) **(** **Figure 7C** **)** and a reduction in chorion melanization (p<0.001) **(** **Figure 7D** **)** that was reminiscent of that observed in PAH knockdown mosquitoes **(** **Figure 6** **).** No change was observed between numbers of eggs laid per female **(** **Figure 7B** **)**. Overall, the similarity of the two phenotypes is consistent with PAH ultimately affecting egg development through the dopamine pathway.

## Discussion

After the ingestion of a blood meal the metabolism of female mosquitoes is challenged with an enormous amount of amino acids in respect to their body weight that must be promptly utilized to support the synthetic processes associated to egg development. Accordingly, many of the genes involved in amino acid metabolism are up-regulated shortly after blood ingestion [36].

Many of the genes involved in amino acid metabolism are highly conserved across distantly related species including vertebrates and invertebrates underlying their importance in nutritional processes. Studies in vertebrates have shown that gene mutations affecting the metabolism of distinct essential and non-essential amino acids are associated with severe multi-organ functional deficits that reduce the life span of the affected individuals and often lead to brain damage [3]. The highly conserved enzyme phenylalanine hydroxylase that converts phenylalanine to tyrosine is the initial enzyme of a key pathway that regulates the synthesis of mosquito neurotransmitters such as dopamine, tyramine and octopamine and the formation of melanin, a complex molecule that play a critical role in the immune response against microbial organism including malaria parasites. Our GC-MS analysis shows that silencing of *PAH* causes a reduction in the levels of tyrosine while inducing a marked accumulation of potentially toxic metabolites such as phenylalanine, and other phenylketones such as phenyllactate and phenylpyruvate. However we did not observe in these mosquitoes a reduction of their life span or behavioural anomalies. Exogenous phenylpyruvate given to mosquitoes in a blood meal produced high mortality immediately after feeding, suggesting that the levels reached upon *PAH*-silencing are not high enough to exert a toxic activity. *PAH*- silencing caused however a marked reduction in egg production and egg maturation in agreement with previous work showing that some amino acids, including phenylalanine are critical for egg production in *Aedes aegypti* and *C. pipiens* [6], [7], [37]. Further, PAH could play a crucial role in vitellogenesis not only in amino acid signalling via the *target of rapamycin* pathway, which in combination with 20- hydroxyecdysone initiates vitellogenesis [4], [5], but also in the assembly of vitellogenin itself that contains one of the highest phenylalanine and tyrosine compositions among all mosquito proteins [38]. In addition *PAH*-silencing caused a significant impairment of the melanin synthesis as shown by a marked reduction of egg chorion melanization after oviposition. The lack of sufficient amount of dopamine in PAH silenced mosquitoes seems to implicate dopamine as the limiting factor for egg chorion melanization. This view was supported by injection of carbidopa, an inhibitor of the decarboxylation of the tyrosine oxidation product DOPA into the dopamine, which induced a phenotype on egg maturation very similar to that observed in *PAH*-silenced mosquitoes. Carbidopa can also inhibit the biosynthesis of serotonin from 5-Hydroxy-L-tryptophan [39] and we cannot exclude the possibility that the small but significant decrease in oviposition rate is also a behavioural effect due to serotonin imbalance. Concomitant to the egg development defects in *PAH*-silenced mosquitoes we observed an impaired ability of the mosquito to melanize *Plasmodium berghei* ookinetes upon the ingestion of an infected blood meal. The melanotic encapsulation of microbial pathogens, a unique feature of insect immunity, also requires a tyrosine precursor which can be obtained by blood meal or endogenously by hydroxylation of phenylalanine into tyrosine [11], [40]. RNAi knockdown experiments in *Aedes aegypti* and *Armigeres subalbatus* demonstrated previously a PAH-dependent melanization response against filarial worms [2], [30]. In contrast, in *A. gambiae*, the mosquito studied here, a knockdown of PAH had no effect on abiotic Sephadex bead melanization [32]. The authors concluded that either the PAH- mediated melanization response to be differently regulated between mosquito species or that it varied between different melanization targets. We have demonstrated that a knockdown of PAH caused a significant increase in phenylalanine and decrease in tyrosine levels in blood-fed *A. gambiae* mosquitoes. Upon infection with *P. berghei* parasites ds*PAH* females were less able to melanize ookinetes, whereas the number of oocysts was not changed. Therefore, we concluded that the endogenous and exogenous amount of tyrosine in *A. gambiae* is insufficient for a fully-functioning melanotic encapsulation response against rodent malaria parasites and requires the hydroxylation of phenylalanine into tyrosine.

We conclude that the hydroxylation of phenylalanine into tyrosine is essential for reproduction and immunity. We also demonstrated that some branches of the phenylalanine metabolism governing oogenesis and oocyte maturation that could not previously be investigated in depth due to low RNAi silencing efficiencies could be targeted using selective enzyme inhibitors.

## Materials and Methods

### Ethics Statement

All animal work was conducted according to UK Home Office Regulations and approved under Home Office License PPL 70/6453.

### *Anopheles* Rearing

The *Anopheles gambiae* G3 strain was reared under standard conditions [41].

### RNA Isolation and cDNA Synthesis

Total RNA was extracted from dissected tissues of 3 mosquitoes at various time points before and after blood meal (3, 24 and 48 h PBM) in Tri-zol (Invitrogen). In RNAi knockdown females, total RNA was extracted from 3 female mosquitoes 24 h post blood meal. First strand cDNA synthesis was performed with 1 µg total RNA, using oligo-d(T) primers (Invitrogen) and Superscript Reverse Transcriptase II (Invitrogen) according the manufacturer’s instructions.

### Quantitative Real- Time PCR

PCR amplification experiments were performed with FAST SYBR Green PCR mix (Applied Biosystems) and analyzed using the ABI Prism 7500 thermocycler according to the manufacturer’s instructions. We used the *PAH* specific primers q-PAH_F 5′-GGATGAGTTTGTGGAGAAGC-3′ and q-PAH_R 5′-CTTGTCGGTCAGGCAGTA-3′ which bind to a region common to all 3 isoforms. Relative expression levels were calculated using the ΔΔCt method as described in Technical Bulletin of the ABI Prism 7500 Manual. The ribosomal protein of RpL19 (AGAP004422), was used for the normalization of the cDNA templates [42]. Three biological repeats were performed and analyzed by t-test.

### dsRNA Production and Injection

The *PAH* target region covering an exon common to all 3 isoforms was amplified by standard PCR from *A. gambiae* cDNA using primers (with flanking *T7* promoter sequence underlined) dsPAH_F (5′-TAATACGACTCACTATAGGGGTCTGCCTGATCTTCTCG-3′) and dsPAH_R (5′-TAATACGACTCACTATAGGGGGCTTCGTTATCCTTGTAGTC-3′) and inserted into the T-easy vector (Promega). PCR products were cleaned up with the QIAquick PCR Purification kit (QIAGEN) dsRNA was synthesized with the MEGAscript T7 Kit (Ambion) and purified using MegaClear kit (Ambion). Its concentration was adjusted to 3 µg/µl and 69 nl were injected into the insect thorax as described [43].

### Metabolic Profiling

2 mosquitoes were extracted in 1 ml ice cold methanol:water (8∶2 v/v) for 2 min. After centrifugation to remove the cellular debris (14000 rpm, 4°C, 15 min) the whole supernatant was transferred to a silanized 1.5 ml glass vial (Agilent Technologies UK Ltd) and dried in a SpeedVac concentrator (Eppendorf). Derivatization was carried out by methoxymation followed by trimethylsilylation using the protocol described by Kind *et al.* [44]. Samples were analyzed on an Agilent 7890 GC coupled to a 5975c mass spectrometer using the Fiehnlib settings [44] and retention-time locking to myristic acid-d27. Deconvolution and integration of the extracted metabolites was performed using the coupled AMDIS-Gavin approach described by Behrends *et al*. [45]. In addition to full scan quantification across the spectrum, phenylalanine, tyrosine, phenyllactate and phenylpyruvate were quantified using selective ion monitoring (SIM). The pathway was based on the Kyoto Encyclopedia of Genes and Genomes [46] and van’t Hof and Sacchari [47]. All experiments were carried out in 4 independent biological replicates.

### Infection by *Plasmodium Berghei*

The *P.berghei*-GFP CON transgenic strain [48] was passaged through CD1 mice and mosquito infections were performed under standard conditions [49]. 4 days after dsRNA injection, mosquitoes were fed on anaesthetised infected mice and the midgut was dissected 8 days later and mounted on glass slides in Vectashield (Vectorlabs). Fluorescent oocysts and melanized ookinetes were counted under a 10x objective of a Nikon TE200 inverted microscope. Combined data from 3 experiments were used to statistically analyze the likelihood of infection using the Fisher’s exact test, the median of *Plasmodium* infection densities using the non-parametric Mann Whitney test of Prism and the proportion of melanized ookinetes using the t-test of the arc-sine transformed proportion.

### Oviposition Assays

Mating was induced by placing about 30 females (3 day old) into a cage with ∼ 200 male mosquitoes (4 day old) and mating couples in the process of copulation were collected in modified plastic falcon tubes as described previously [50]. Mated females were reared collectively and females were injected at 24 h post-mating with ds*PAH* or ds*LacZ* RNA. At 3 days after injection the mosquitoes were blood-fed. Blood-fed females were placed into single plastic cups aligned with 5 cm filter paper strip and filled with 50 ml larval rearing water [50]. The likelihood of oviposition was calculated using the Fisher’s exact test, the numbers of eggs laid and arc-sine transformed hatching rates were analyzed using the t-test.

### Carbidopa Injection

Mating couples were collected as above and blood-fed 24 h later. In solution carbidopa has a short half-life of 1–2 h. Therefore, in order to investigate the effect of carbidopa on ovipostion and egg maturation fed females were injected at a late stage of oogenesis (∼53 h post blood meal) into the thorax either with PBS solution or 160 mM carbidopa (Sigma) in PBS and allowed to recover from injection for 2 h before being placed into oviposition cups for egg laying in 4 independent experiments.The egg melanization rate was measured from eggs laid by a total of 24 PBS and 9 carbidopa injected females from 2 independent experiments. The arc-sine transformed melanization rates were analyzed using the t-test.

### Survival Analysis

In all experiments, the survival of a minimum of 40 3–4 day old females was recorded daily from time point of ingestion of a mouse blood meal until 7–10 days after the blood meal. Phenylpyruvate was added to human blood meal and administered to mosquitoes via a membrane feeder. Where females obtained repeated blood meals, blood meals were given every 3 days and survival was recorded from the time when females had their first blood meal. Only fully-fed females were included in the survival analysis and they did not have access to an egg collection device in order to prevent deaths caused by drowning. Survival rates at the various time points were averaged between biological replicates (minimum of 3) and the standard error of the mean was displayed. All survival data were analyzed using the log-rank test of Prism (GraphPad Software Inc.).

## Supporting Information

Figure S1 **Multiple alignment of PAH protein sequences of** ***A. gambiae*** **.** The 3 Ag*PAH* transcripts are translated into the 3 different proteins: AGAP005712-PA (XP_001688715.1), AGAP005712-PC (XP_315721.4) and AGAP005712-PB (XP_315722.4). The beginning of the regulatory, catalytic, and tetramerization domains are indicated with ▸, *,>respectively [30]. The shaded area represents the region used for dsRNA synthesis.
(TIF)
