# Supplementary figures and images for: Phenylalanine Metabolism Regulates Reproduction and Parasite Melanization in the Malaria Mosquito
Source: PLoS One. 2014 Jan 7;9(1):e84865. doi: 10.1371/journal.pone.0084865 (PMC3883676; doi:10.1371/journal.pone.0084865)

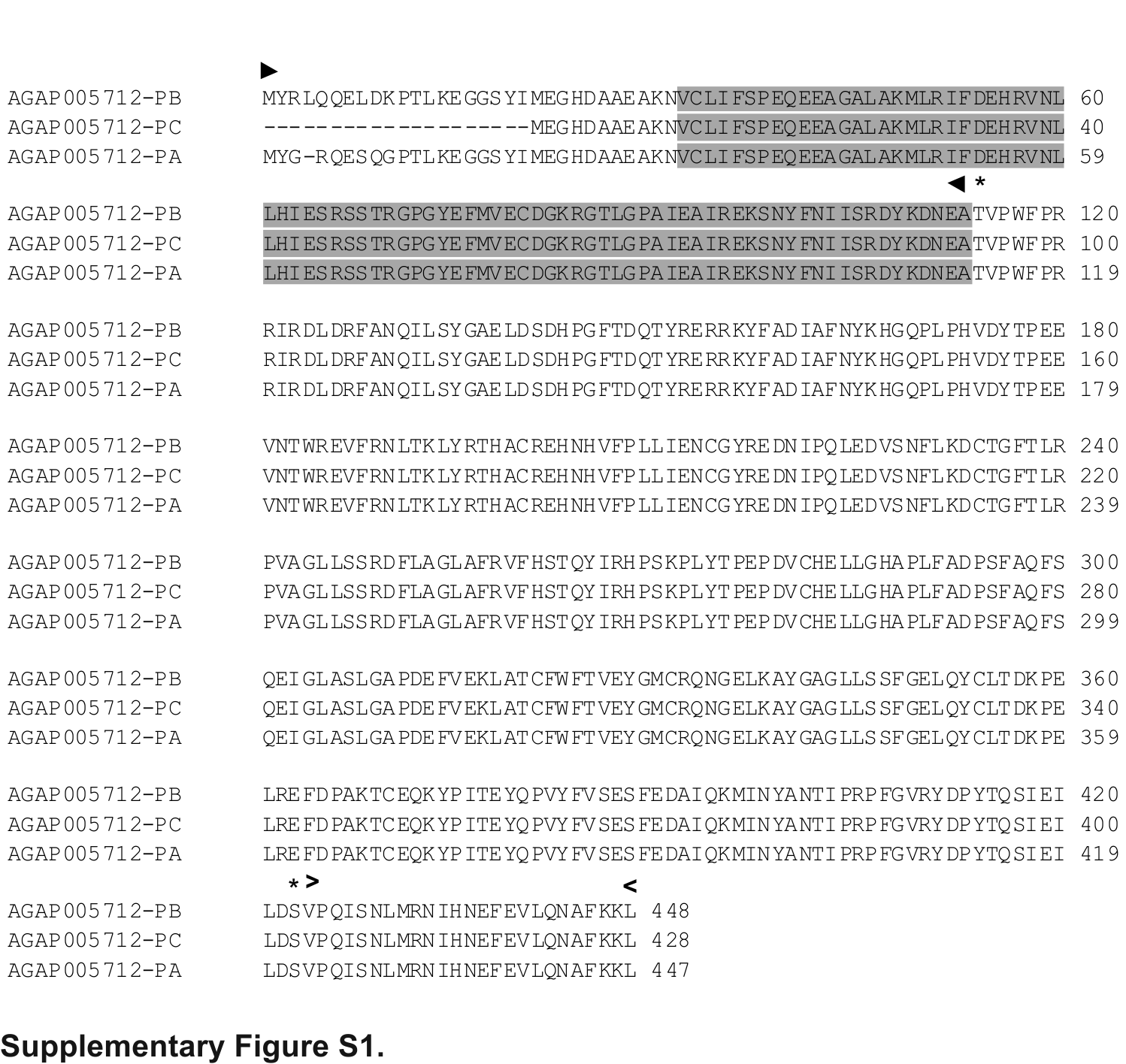

Supplement: Figure S1 — Multiple alignment of PAH protein sequences of A. gambiae . The 3 AgPAH transcripts are translated into the 3 different proteins: AGAP005712-PA (XP_001688715.1), AGAP005712-PC (XP_315721.4) and AGAP005712-PB (XP_315722.4). The beginning of the regulatory, catalytic, and tetramerization domains are indicated with ▸, *,>respectively [30]. The shaded area represents the region used for dsRNA synthesis. (TIF) [file pone.0084865.s001.tif]
